# Fast, faster, and the fastest structured illumination microscopy

**DOI:** 10.1038/s41377-024-01505-2

**Published:** 2024-08-12

**Authors:** Tianyu Zhao, Ming Lei

**Affiliations:** MOE Key Laboratory for Nonequilibrium Synthesis and Modulation of Condensed Matter, School of Physics, Xi’an Jiaotong University, Xi’an, 710049 China

**Keywords:** Super-resolution microscopy, Imaging and sensing

## Abstract

Parallel acquisition-readout structured-illumination microscopy (PAR-SIM) was designed for high-speed raw data acquisition. By utilizing an xy-scan galvo mirror set, the raw data is projected onto different areas of the camera, enabling a fundamentally stupendous information spatial-temporal flux.

Super-resolution microscopy, with representations of stimulated emission depletion (STED) microscopy, photo-activated localization microscopy (PALM), stochastic optical reconstruction microscopy (STORM), and structured-illumination microscopy (SIM), has drawn considerable interest among biologists, as they are able to surpass the fundamental diffraction limit and visualize the fine details of intracellular structures^1–3^. Among these techniques, SIM doubles the spatial resolution of wide-field fluorescence microscopes while enabling high-speed imaging with minimizing photodamage, which is in high demand in living-cell imaging. A variety of new biological phenomena have been recorded with SIM such as fragmentation of the migrasomes^4^, DNA damage^5^, and protein crystal growth^6^, thus providing biologists with a powerful tool for observing subcellular organelle interactions^7^.

In SIM, the periodic illumination down-modulates the high spatial frequency of the sample information in the Fourier domain. Several raw images with different pattern phases and orientations are captured to undo the frequency modulation. The final high-resolution image is computed after separating and recombining the spectral information^8,9^. In the past decades, various devices have been introduced to SIM to further enhance its spatial-temporal resolution. The ferroelectric liquid crystal spatial light modulator with a 4 kHz fresh rate is widely used to replace the traditional mechanical movement grating^10^. Galvanometers conjoined with an electro-optic modulator is another way to flexibly and quickly control the phase and direction of structured illumination patterns, and increase the super-resolution reconstruction frame rate to 25 Hz^11^. The latest 3D-SIM further improves the imaging speed to 24 frame/s in single-layer 3D-SIM which is based on the digital micromirror device with a 23 kHz switching speed^12^. However, the speed of SIM is also limited by the detector. Researchers have to tradeoff between the exposure-readout speed of the sCMOS (scientific complementary metal-oxide-semiconductor) camera, resulting in a low signal-to-noise ratio and narrow visual fields.

In a recently published paper in *Light: Science & Applications*, Peng Xi’s team from Peking University proposed a new approach to elevate SIM frame rates called parallel acquisition-readout SIM (PAR-SIM), as shown in Fig. 1 (ref. ^13^). The key innovation in their approach is utilizing a galvo mirror set to actively project the raw data of SIM onto six different reigns of the sCMOS detector. They also effectively exploit the camera with both exposure and readout region by using the light-sheet mode instead of the normal area mode. Thus PAR-SIM can theoretically achieve 6× higher SIM frame rates without sacrificing SNR (signal-to-noise ratio).

**Fig. 1:**
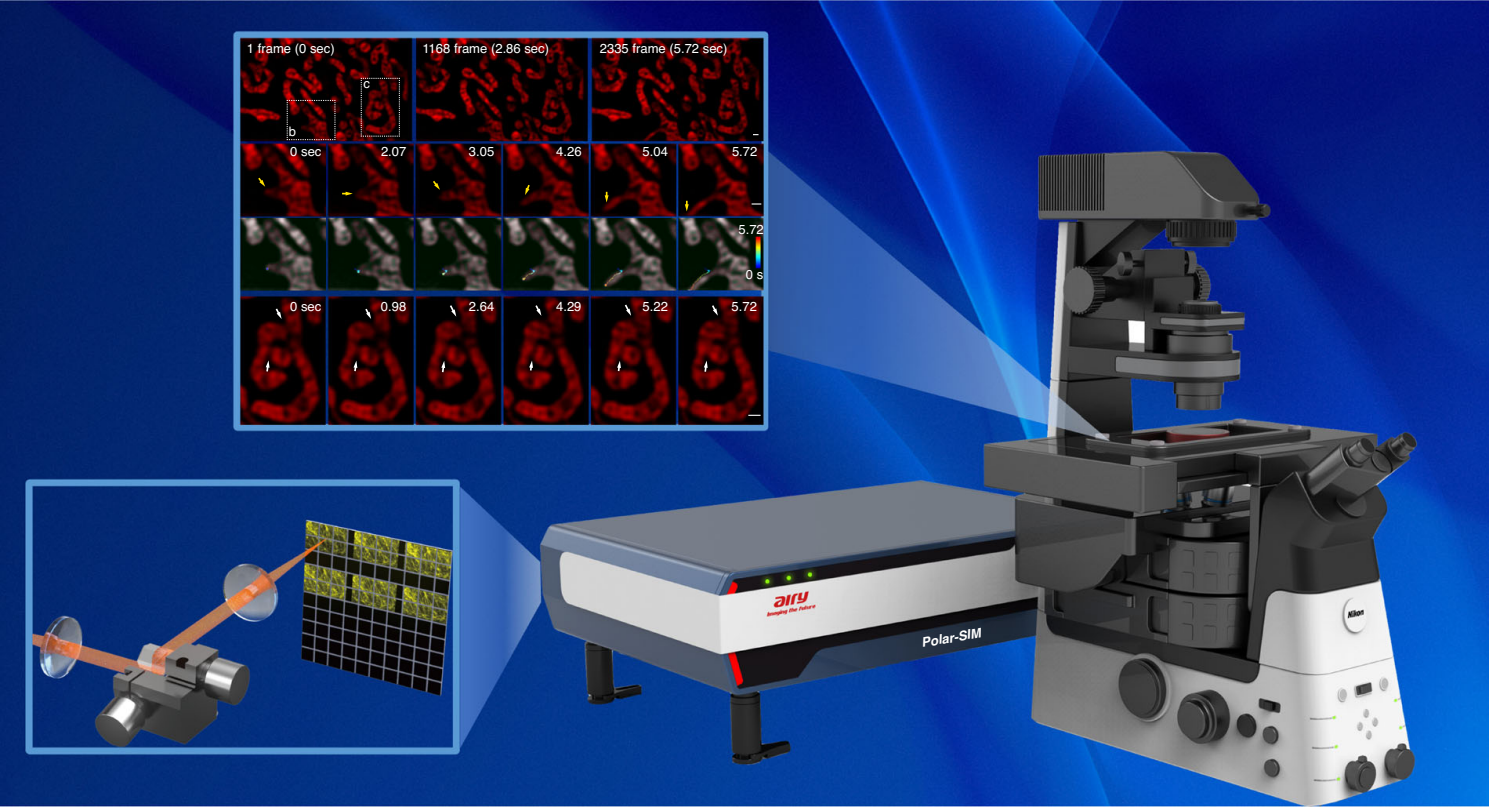
PAR-SIM enables a fundamentally stupendous information spatial-temporal flux

The performance of the PAR-SIM is demonstrated in both fixed model organelles and live mitochondrial interaction. The 100 nm fluorospheres serve as a resolution ruler, whose FWHM (full width at half maxima) is 258 nm in WF (wide field) and 99 nm in PAR-SIM. The high-speed imaging capability is evaluated through the fast mitochondrial dynamic tubulation. 10 s video with 408.16 Hz super-resolution image framerate is generated at 0.4 ms exposure time and 3-rolling reconstruction strategy. They further improve the reconstruction algorithm with iteratively optimized phase difference estimates and prior knowledge, ensuring precise SIM reconstruction at ultralow exposure time. The results consistently indicated a substantial enhancement in resolution and fidelity, while improving the speed significantly. In addition, this parallel exposure-readout mode can integrate with other techniques such as single-molecule localization microscopy, SOFI(super-resolution optical fluctuation imaging), Fourier ptychographic microscopy, label-free imaging, etc. to further accelerate raw data acquisition, where the same sCMOS rolling shutter mode is employed.

The immediate follow-up work would be to improve the reconstruction speed. Coupled with time-consuming postprocessing procedures, it can be challenging to locate useful data before waiting for the super-resolution results^14^. Although some effort was made to achieve the real-time reconstruction, the step of parameter estimation is not counted and a better parameter estimation algorithm is required to improve the accuracy of reconstructed images^15,16^. In addition, since the hardware performance is stretched to the limit, reducing the number of raw images would make the possibility for realizing the fastest SIM. A theoretical framework is introduced in Florian’s work, which demonstrated that 4 image acquisitions are enough to obtain super-resolution in SIM^17^. This may enable even faster cellular dynamics, such as the spreading of Ca^2+^ nanodomains originating from a single Ca^2+^ channel and the propagation of membrane potentials along axons to be captured in high definition^18^. However, SIM is always prone to reconstruction artifacts, because the reconstruction process is essentially an ill-posed inverse problem. More robust reconstruction algorithms are required for better fidelity^19,20^. In the future, with the novel hardware and credible algorithms, we can expect SIM could replace the existing conventional fluorescent microscopy and be promoted as a daily imaging tool.
